# Construction of a genetic map using EST-SSR markers and QTL analysis of major agronomic characters in hexaploid sweet potato (*Ipomoea batatas* (L.) Lam)

**DOI:** 10.1371/journal.pone.0185073

**Published:** 2017-10-11

**Authors:** Jin-Hee Kim, Il Kyung Chung, Kyung-Min Kim

**Affiliations:** 1 School of Applied Biosciences, College of Agriculture & Life Sciences, Kyungpook National University, Daegu, Korea; 2 Department of Biotechnology, Catholic University of Daegu, Gyeongsan-Si, Gyeongbuk, Korea; Huazhong University of Science and Technology, CHINA

## Abstract

The Sweet potato, *Ipomoea batatas* (L.) Lam, is difficult to study in genetics and genomics because it is a hexaploid. The sweet potato study not have been performed domestically or internationally. In this study was performed to construct genetic map and quantitative trait loci (QTL) analysis. A total of 245 EST-SSR markers were developed, and the map was constructed by using 210 of those markers. The total map length was 1508.1 cM, and the mean distance between markers was 7.2 cM. Fifteen characteristics were investigated for QTLs analysis. According to those, the Four QTLs were identified, and The LOD score was 3.0. Further studies need to develop molecular markers in terms of EST-SSR markers for doing to be capable of efficient breeding. The genetic map created here using EST-SSR markers will facilitate planned breeding of sweet potato cultivars with various desirable traits.

## Introduction

Sweet potato [*Ipomoea batatas* (L.) Lam.] is a hexaploid (2n = 6x = 90) plant that belongs to the family Convolvulaceae [1,2]. Sweet potato is a crop that could provide a valuable source of nutrition in many areas [3]. Additional varieties of sweet potato are desirable; however, despite its agroeconomic importance, this hexaploid crop is difficult to breed owing to the complexity of its genetics and the lack of genomic resources [4,5]. Genetic markers offer a number of avenues for genetic improvement of the sweet potato. Randomly amplified polymorphic DNA (RAPD) analysis, which was developed in 1990 [6,7], is a powerful molecular marker technique for genetics and plant breeding [8]. However, it is also expensive, labor intensive, and time consuming to develop genomic SSR markers. In contrast, EST-SSRs can be rapidly developed from an EST database, at lower cost. Moreover, EST-SSRs can facilitate direct gene tagging for quantitative trait locus (QTL) mapping of traits of agronomic importance and increase the efficiency of marker-assisted selection [9]. In addition, EST-SSRs show a higher level of transferability to closely related species than do genomic SSR markers [10–12] and can serve as anchor markers for comparative mapping and evolutionary studies [13]. Breeding practices for improving the quality of sweet potato are ongoing worldwide, and the molecular markers recently developed for sweet potato have demonstrated good potential to be used in genetic selection [14]. Genetic mapping studies, which are the basis for analyzing sweet potato genomes and molecular breeding, have not yet been conducted, although several papers have been published that have produced gene maps using RAPD [15], AFLP [4,5], and AFLP and SSR markers [16]. Since sweet potatoes are a crop that guarantees a high yield in a harsh environment, cultivating additional varieties could help to solve food, energy, and environmental problems. Although sweet potato is one of the seven major food crops in the world, it is a hexaploid (2n = 6x = 90), the dielectric is large and the composition of the polyploid is complicated. Therefore, few investigations of its genetics and genomics have been performed domestically or internationally. Establishing a research base for identification of genes underlying important traits could facilitate breeding for increased pest resistance and other valuable traits. In this study, we developed EST-SSR markers for sweet potato, and constructed a genetic map of this hexaploid crop, which we used to perform QTL analysis.

## Materials and methods

### Plant materials and construction of the genetic map

The sweet potato cultivar Sinwhangmi was used for development of the EST-SSR marker [17] genetic mapping was based on 137 populations, which are generated by means of crossing between Yeseumi, is known for high yield ability, high resistance to *Fusarium oxysporumas* and as domestic cultivar, and Annobeny, is known as Japanese cultivar by yellow and sweet root in addition to low yield ability. The populations got form the National Institute of Crop Science in Muan. Linkage analysis and map construction were used for identify 245 EST-SSR markers. Markers were associated at a LOD score of 3.0. Grouping of markers and map construction were performed using Mapmaker version 3.0 and Mapchart version 2.2.

### Observation of major agronomic characteristics

A total of 15 major agronomic characteristics of sweet potatoes were investigated according to the method described by the Korea Seed & Variety Service (Table A in S1 File). We selected sweet potato roots of medium size and the greatest possible variety of shapes. The sweet potato circled in the figure was selected for analysis (Figure A (A) in S1 File). Root form was classified into 5 types: ovate, oval, obovate, cylindrical, and indeterminate (Figure A (B) in S1 File). The sweet potato was cut in the middle, and the skin thickness was measured at three locations using a Digimatic caliper (Mitutoyo Company, Japan). The depth of root buds was classified as shallow, medium, and deep (Figure A (C, D) in S1 File). The ratio of length to width was measured. The color of the skin and the flesh were measured at three points using a color meter (Tes-135, USA) (Figure A (E, F) in S1 File). The length of the internode was measured 3 times at the one-third point of the main stem. The internode diameter was measured 3 times at the midpoint of the main stem (Figure B in S1 File). Number of leaf lobes was classified as none, three, five, and seven. Four types of unlobed leaf shapes were identified: cordate, triangular, reniform, and round. Leaf lobe depth was classified as very shallow, shallow, middle, deep, and very deep (Figure C in S1 File). The degree of anthocyanin biosynthesis in the burl and terminal buds was classified as none, medium, and strong (Figure D in S1 File). The frequency distribution of the investigated characteristics was analyzed using SigmaPlot (SPSS Science, Chicago, IL).

### Quantitative trait loci (QTL) analysis

QTL analysis of a total of 15 major sweet potato cultivated traits was performed using the Composite Interval Mapping (CIM) function of the WinQTL Cartographer 2.5 program. The LOD (logarithm of odds) score was 3.0. This program requires several factors such as the genetic distance between each marker, names of all markers, number of chromosomes, genotyping data, and values of target traits.

## Results

We constructed a genetic-linkage map using 210 of the 245 identified markers. The total length was 1508.1 cM and the mean distance between markers was 7.2 cM. In this study, 15 characteristics were investigated, and QTLs were identified for 4 of these characteristics. The LOD score was 3.0. The internode length, main color of the outer skin, and secondary color of the outer skin were found to originate from the mother plant (Yeseumi), and skin thickness was confirmed to be influenced by the father plant (Annobeny). QTL analysis of the identified characteristics indicated that QTLs for the main color of the outer skin were distributed in various groups; 3 QTLs were identified in genetic-linkage group 14. Two or more QTLs were identified in linkage groups 4, 7, 8, and 13. No QTLs were identified in linkage groups 2, 5, 10, 11, and 15. These results will facilitate marker-assisted selection for breeding of sweet potato varieties, permitting identification of desired traits through planned breeding.

### Construction of the genetic map using EST-SSR markers

A total of 245 EST-SSR markers selected for polymorphism and were used for gene mapping (accession number; LC322425-LC322922, entry ID; 59bd35e7028ed6b8b2000046.s2-59bd35e7028ed6b8b2000046.s711, http://ddbj.nig.ac.jp). Using a population resulting from crossing of the Yeseumi and Annobeny samples, a genetic map was constructed using 210 of the 245 markers. The total map length was 1508.1 cM and the mean distance between markers was 7.2 cM. The linkage groups differed in the number of markers per group, but the markers were evenly distributed throughout the genome (Table 1 and Figure E in S1 File).

**Table 1:** Distribution and distance of markers by group.

Group number	No. of mapping markers	Distance (cM)
1	25	121.9
2	21	81.4
3	14	98.8
4	16	100.9
5	18	101.7
6	11	91.0
7	19	107.6
8	7	66.8
9	11	146.1
10	9	75.7
11	12	108.0
12	12	123.3
13	11	114.7
14	15	100.3
15	9	69.9
Subtotal	210	-
Others^a^	35	-
Total	245	1508.1
Average^b^	-	7.2

^a^ Others: This number was not grouping.

^b^ Average: Interval between makers in genetic map.

### Observation of the major agronomic characteristics

The average internode length (cm) of the mother and father plants was 2.8 ± 0.4, and 4.5 ± 0.2, respectively. The range of the population was 1.1–4.4 and the mean was 2.0 ± 0.6. The average internode diameter (mm) of the mother and father plants was 3.4 ± 0.2 and 3.8 ± 0.5, respectively. The range of the population was 2.7–7.0 and the mean was 4.0 ± 0.7. The degree of anthocyanin biosynthesis in the terminal buds of the mother and father plants was 1.0 ± 0.0 and 2.0 ± 0.0, respectively. The range of the population was 1.0–3.0 and the mean was 2.0 ± 0.7. The degree of anthocyanin expression in the burl of the mother and father plants was 1.0 ± 0.0, and 2.0 ± 0.0, respectively. The range of the population was 1.0–3.0 and the mean was 1.0 ± 0.6. The number of leaf lobes of the mother and father plants was 7.0 ± 0.0 and 1.0 ± 0.0, respectively. The range of the population was 1.0–5.0 and the mean was 3.0 ± 1.6. For unlobed leaf number, the average for the father plants was 1.0 ± 0.0. The range of the population was 1.0–2.0 and the mean was 2.0 ± 0.5. The average number of leaf lobes of the mother plants was 5.0 ± 0.0. The range of the population was 1.0–7.0 and the mean was 4.0 ± 1.9. The average root shape of parent was 1.0 ± 0.0. The range of the population was 1.0–5.0 and the mean was 2.0 ± 1.6. The average length to width ratios of the mother and father plants were 2.4 ± 0.3 and 3.3 ± 0.2, respectively. The range of the population was 1.1–7.9 and the mean was 4.0 ± 1.2. The average skin thickness (mm) of the mother and father plants was 1.9 ± 0.4 and 3.3 ± 0.3, respectively. The range of the population was 1.1–4.6 and the mean was 3.0 ± 0.6. The average main color of the outer skin of the mother and father plants was 76.6 ± 2.1 and 67.3 ± 2.0, respectively. The range of the population was 50.6–87.4 and the mean was 76.0 ± 6.7. The average secondary color of the outer skin of the mother and father plants was 74.7 ± 3.0 and 66.3 ± 5.0, respectively. The range of the population was 60.2–85.4 and the mean was 76.0 ± 5.1. The main color of the flesh of the mother and father plants was 64.4 ± 2.9 and 70.8 ± 2.2, respectively. The range of the population was 55.8–102.0 and the mean was 73.0 ± 7.8. The secondary color of the flesh of the mother and father plants was 73.7 ± 2.9, and 80.7 ± 2.4, respectively. The range of the population was 52.9–113.0 and the mean was 84.0 ± 12.6. The average depth of root buds of the mother and father plants was 2.0 ± 0.0 and 1.0 ± 0.0, respectively. The range of the population was 1.0–3.0 and the mean was 2.0 ± 0.7 (Table 2).

**Table 2:** Major agronomic characteristics.

Item	Characters		Parents			Population	
			Mother	Father	Means	Range	Means
Above-ground part	Stem	Length of internode (cm)	2.8 ± 0.4^a^	4.5 ± 0.2	3.6 ± 0.9	1.1–4.4	2.0 ± 0.6
		Internode diameter (mm)	3.4 ± 0.2	3.8 ± 0.5	3.6 ± 0.4	2.7–7.0	4.0 ± 0.7
		Degree of anthocyanin biosynthesis in terminal bud	1.0 ± 0.0	2.0 ± 0.0	1.5 ± 0.5	1.0–3.0	2.0 ± 0.7
		Degree of anthocyanin biosynthesis in burl	1.0 ± 0.0	2.0 ± 0.0	1.5 ± 0.5	1.0–3.0	1.0 ± 0.6
	Leaf	Number of lobes per leaf	7.0 ± 0.0	1.0 ± 0.0	4.0 ± 3.0	1.0–5.0	3.0 ± 1.6
		Unlobed leaf shape	–^b^	1.0 ± 0.0	–	1.0–2.0	2.0 ± 0.5
		Depth of leaf lobe (mm)	5.0 ± 0.0	–	–	1.0–7.0	4.0 ± 1.9
Underground part	Root	Root form (shape)	1.0 ± 0.0	1.0 ± 0.0	1.0 ± 0.0	1.0–5.0	2.0 ± 1.6
		Length to width ratio	2.4 ± 0.3	3.3 ± 0.2	2.9 ± 0.5	1.1–7.9	4.0 ± 1.2
		Skin thickness (mm)	1.9 ± 0.4	3.3 ± 0.3	2.6 ± 0.8	1.1–4.6	3.0 ± 0.6
		Main color of outer skin (△E)	76.6 ± 2.1	67.3 ± 2.0	72.0 ± 5.1	50.6–87.4	76.0 ± 6.7
		Secondary color of outer skin (△E)	74.7 ± 3.0	66.3 ± 5.0	70.5 ± 5.9	60.2–85.4	76.0 ± 5.1
		Main color of flesh (△E)	64.4 ± 2.9	70.8 ± 2.2	67.6 ± 4.1	55.8–102.0	73.0 ± 7.8
		Secondary color of flesh (△E)	73.7 ± 2.9	80.7 ± 2.4	77.2 ± 4.4	52.9–113.0	84.0 ±12.6
		Depth of root buds(mm)	2.0 ± 0.0	1.0 ± 0.0	1.5 ± 0.5	1.0–3.0	2.0 ± 0.7

^a^ Mean ± standard deviation.

b–: Not applicable.

For internode length of internode, the mother and father plants were to the right of the distribution; for internode diameter both the mother and father plants were to the left of the distribution. In the degree of anthocyanin biosynthesis in terminal bud, mother plant was not on the distribution and the father plant was in the middle of the distribution. In the degree of anthocyanin biosynthesis in the burl, the mother plant was not on the distribution and the father plant was in the middle of the distribution (Figure F in S1 File). For number of leaf lobes, mother plants showed zero lobes and the father plants showed 5 leaf lobes. Father plants showed cordate leaves. Mother plants showed deep leaf lobes, and father plants showed very shallow leaf lobes. Mother and father plants showed ovate root form (Figure G in S1 File). For length to width ratio, mother and father plants were to the right of the distribution. For skin thickness, the mother plant was to the left of the distribution and the father plant was to the right of the distribution. For the main color of the outer skin, the mother plant to the right of the distribution and father plants were to the left of the distribution. For the secondary color of the outer skin, the mother plant was to the right of the distribution and the father plant to the left of the distribution (Figure H in S1 File). For the main color of the flesh, both mother and father plants to the left of the distribution. For the secondary color of the flesh, the mother and father plants were to the left of the distribution. Mother plants showed medium depth of root buds and father plants showed shallow root bud depth (Figure I in S1 File).

### QTL analysis of agronomic characteristics

#### Length of internode (cm)

In genetic-linkage group 4, qLi8 was located between s25-1-1 to s1472-1. The LOD score was 3.63. The R2 value was 0.16. Yeseumi increased the effect (Figure J in S1 File). In genetic-linkage group 7, qLi7 was located at s13261-3-1. The LOD score was 4.13. The R2 value was 0.10. Yeseumi increased the effect (Figure K in S1 File). In genetic-linkage group 8, qLi4 was located between s1328-1 and s463-2. The LOD score was 3.07. The R2 value was 0.08. Yeseumi increased the effect (Figure L in S1 File).

#### Skin thickness (mm)

Genetic-linkage group 4 was associated with QTLs for skin thickness (mm) of the Yeseumi/Annobeny population. qST4 was located between s1267-1 and s439-1. The LOD score was 4.65 and the R2 value was 0.05. Annobeny increased the effect (Figure M in S1 File).

#### Bare skin main color (△E)

A total of 9 linkage groups, groups 1, 3, 6, 7, 8, 9, 12, 13, and 14, were associated with QTLs for the main color of the outer skin of the Yeseumi/Annobeny population. In genetic-linkage group 1, qBsmc1 was located between s61-1 and s1671-2, and in genetic-linkage group 3, qBsmc3 was located between s1301-2-2 and s999-1-2. The LOD scores were 5.40 and 3.62, respectively. The R2 values were 0.01 and 0.32, respectively. Yeseumi increased the effect (Figure N in S1 File and Figure O in S1 File). In group 6, qBsmc6-1 and qBsmc6-2 were located between s1680-1 and s571-2, s571-2 and s207-1, respectively. The LOD scores were 4.09 and 3.94, respectively. The R2 values were 0.01 and 0.01, respectively. Yeseumi and Annobeny, respectively, increased the effect (Figure P in S1 File). In group 7, qBsmc7-1 and qBsmc7-2 were located between s1685-2 and s61-2, s10-2 and s422-2-2, respectively. The LOD scores were 3.13 and 4.28. The R2 values were 0.25 and 0.25, respectively. Yeseumi increased the effect (Figure Q in S1 File). In group 8, qBsmc8-1 and qBsmc 8–2 were located between s1328-1 and s463-2, s463-2 and s1191-2-2, and the LOD scores were 3.06 and 3.65, respectively. The R2 values were 0.65 and 0.95, respectively. Yeseumi increased the effect (Figure R in S1 File). In group 9, qBsmc9-1 and qBsmc9-2 were located between s1191-3-2 and s996-2-2, s2-2 and s71-4, respectively. The LOD scores were 4.31 and 6.82. The R2 values were 0.01 and 0.93, respectively. Yeseumi increased the effect (Figure S in S1 File). In group 12, qBsmc12 was located between s1437-4 and s1437-2, and the LOD score was 3.25. The R2 value was 0.01. Yeseumi increased the effect (Figure T in S1 File). In group 13, qBsmc13 was located between s678-2 and s42-4. The LOD score was 4.54. The R2 value was 0.01. Annobeny increased the effect (Figure U in S1 File). In group 14, qBsmc14-1, -2, and -3 were located between qUI13 was located between s367-3 and s639-1-1, s3639-1-1 and s1685-1, s277-2 and s428-2 respectively. The LOD scores were 4.50, 3.99, and 3.03. The R2 values were 0.78, 0.74, and 0.01, respectively. Yeseumi increased the effect (Figure V in S1 File).

#### Bare skin secondary color (△E)

In genetic-linkage group 6, qBssc6 was located between s1680-1 and s571-2. The LOD score was 3.49. The R2 value was 0.01. Yeseumi increased the effect (Figure W in S1 File). qBssc13 was located between s678-2 and s42-4. The LOD score was 3.22. The R2 value was 0.54. Yeseumi increased the effect (Figure X in S1 File).

**Fig 1:**
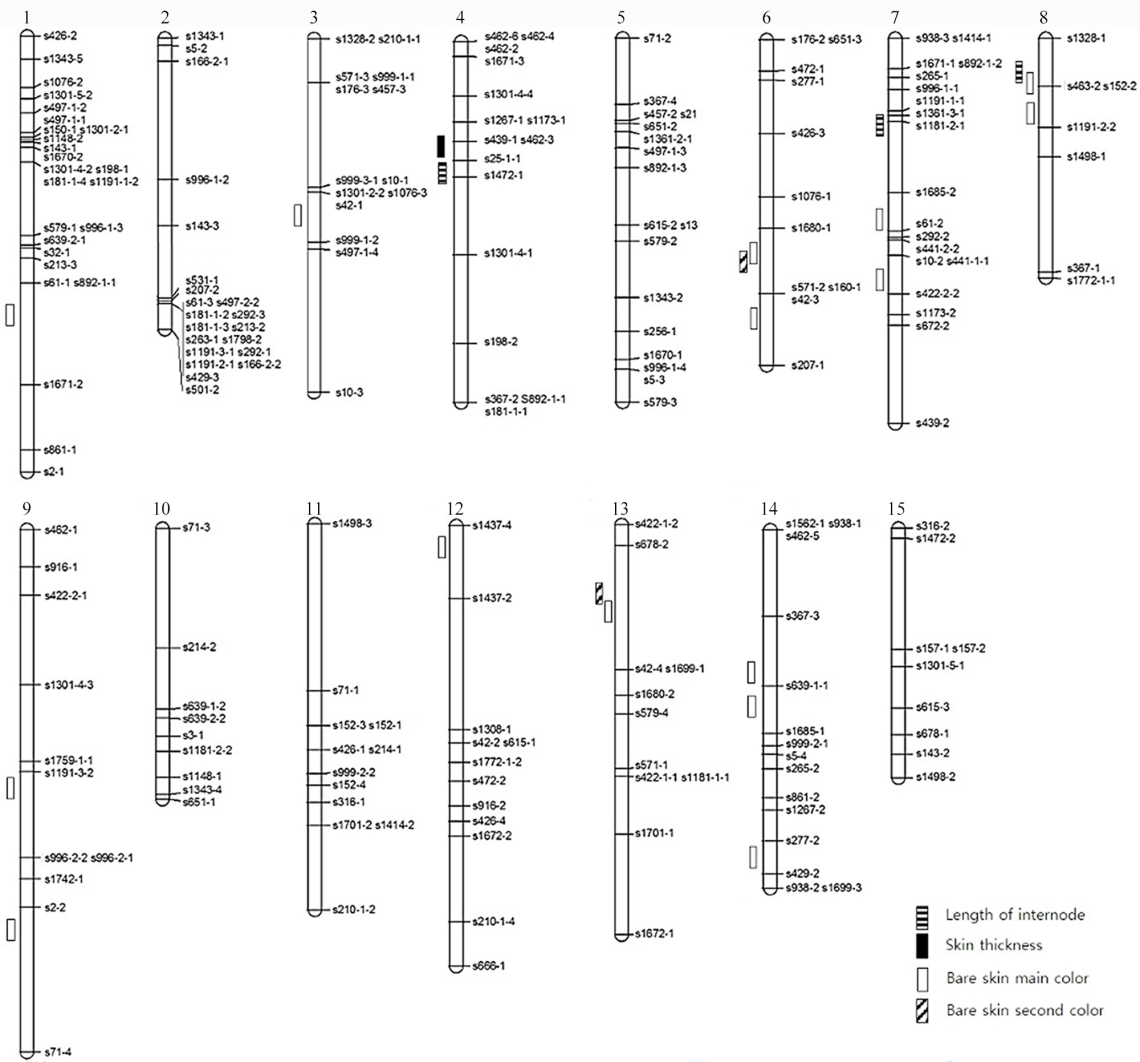
Genetic map and QTL mapping associated with sweet potato.

QTL analysis of the identified characteristics indicated that QTLs for the main color of the outer skin were distributed in various groups; 3 QTLs were identified in genetic-linkage group 14. Two or more QTLs were identified in linkage groups 4, 7, 8, and 13. No QTLs were identified in linkage groups 2, 5, 10, 11, and 15 (Fig 1).

## Discussion

Although marker-assisted mapping has been performed for many crops, no such studies are available for sweet potato. Ukoskit and Thompson [15] reported that sweet potato shows polysomic inheritance, based on the segregation ratio and genetic linkage relationships of RAPD markers. RAPD linkage maps of the Vardaman and Regal cultivars were constructed. The Vardaman map had a predicted coverage of 10.5% at a 25 cM interval, with a genome size of 5024 cM. For the Regal cultivar, genome coverage was estimated to be 5.6% at a 25 cM interval, with a genome size of 6560 cM. The average length is estimate at 56 to 73 cM [15]. Kriegner et al. [4] constructed an AFLP-based linkage map of the cultivars Tanzania and Bikilamaliya. A total of 632 (Tanzania) and 435 (Bikilamaliya) AFLPs were found to be distributed among 90 and 80 linkage groups, respectively. Total map lengths were 3655.6 cM and 2011.5 cM, respectively, with an average distance of 5.8 cM between adjacent markers [4]. In this study, we constructed a genetic linkage map based on EST-SSR markers using the Yeseumi and Annobeny cultivars. The map was constructed using 210 of the total of 245 identified markers. The total length was 1508.1 cM and the mean distance between markers was 7.2 cM (Table 1). The number of markers per group differed, but markers were evenly distributed throughout. Zhao et al. [16] reported identification of 90 linkage groups and mapped a total of 27 QTLs for dry-matter content, explaining 9.0–45.1% of the variation. Cervantes-Flores et al. [18] identified QTLs for dry matter, starch, and β-carotene content in a hexaploid sweet potato mapping population derived from a cross between Tanzania, a white-fleshed. In both parental maps, QTL analysis revealed the presence of 13 QTLs for storage root dry-matter content, 12 QTLs for starch content, and 8 QTLs for β-carotene content. In this study, 15 characteristics were investigated, and QTLs were identified for 4 characteristics. The LOD score was 3.0. The internode length, main color of the outer skin, and the secondary color of the outer skin were found to originate from the mother plant (Yeseumi), and skin thickness was confirmed to be influenced by the father plant (Annobeny) (Table 3). Rice, which is the largest food crop in the world, has long been the subject of intense agronomic interest. A genetic map of rice was constructed by McCouch et al. [19]. In 2005, the complete nucleotide sequence of the rice genome was published [20]. Although rice genetics has been studied for over a century, recent advances in large-scale mutagenesis experiments and sequencing of expressed sequence tags (ESTs), full-length cDNAs, and publication of the genomes of both *Oryza sativa* ssp. *japonica* and *O*. *sativa* ssp. *indica* have significantly added to our understanding of gene networks, gene function, and allelic and sequence diversity. In addition, the conventions for naming of genes and alleles have become based on biological traits [21]. Sweet potato is another globally important food crop, and further studies using molecular markers, especially EST-SSR markers, will be needed in the future. Moreover, in addition to acting as molecular tags for particular traits, EST-SSR markers can also be used to identify corresponding genes. Thus, development of EST-SSR markers will facilitate efficient molecular analysis of sweet potato. The genetic map created here using EST-SSR markers will act as a foundation for further study of sweet potato breeding. These data will help in development of high-quality sweet potato cultivars to overcome the agricultural challenges created by climate change and other unfavorable conditions.

**Table 3:** Quantitative trait loci (QTL) analysis.

Characters	Group Number	Locus	LOD	Add.^a^	R^2^ ^b^	Marker bordering the QTL^c^	Increasing effect^d^	Closed marker
Length of internode (cm)	4	qLi4	3.63	1.12	0.16	s25-1-1~s1472-1	Yeseumi	s1472-1
	7	qLi7	4.13	1.06	0.10	s1361-3-1	Yeseumi	s1361-3-1
	8	qLi8	3.07	1.13	0.08	s1328-1~s463-2	Yeseumi	s1328-1
Skin thickness (mm)	4	qSt4	4.65	-0.33	0.05	s1267-1~s439-1	Annobeny	s439-1
Main color of outer skin (△E)	1	qBsmc1	5.40	9.50	0.01	s61-1~s1671-2	Yeseumi	s1671-2
	3	qBsmc3	3.62	9.88	0.32	s1301-2-2~s999-1-2	Yeseumi	s1301-2-2
	6	qBsmc6-1	4.09	9.09	0.01	s1680-1~s571-2	Yeseumi	s1680-1
	6	qBsmc6-2	3.94	-3.38	0.01	s571-2~s207-1	Annobeny	s571-2
	7	qBsmc7-1	3.13	11.35	0.25	s1685-2~s61-2	Yeseumi	s1685-2
	7	qBsmc7-2	4.28	11.95	0.25	s10-2~s422-2-2	Yeseumi	s422-2-2
	8	qBsmc8-1	3.06	10.80	0.65	s1328-1~s463-2	Yeseumi	s1328-1
	8	qBsmc8-2	3.65	11.38	0.95	s463-2~s1191-2-2	Yeseumi	s463-2
	9	qBsmc9-1	4.31	9.07	0.01	s1191-3-2~s996-2-2	Yeseumi	s1191-3-2
	9	qBsmc9-2	6.82	10.50	0.93	s2-2~s71-4	Yeseumi	s71-4
	12	qBsmc12	3.25	10.98	0.01	s1437-4~s1437-2	Yeseumi	s1437-4
	13	qBsmc13	4.54	-8.18	0.01	s678-2~s42-4	Annobeny	s678-2
	14	qBsmc14-1	4.50	10.32	0.78	s367-3~s639-1-1	Yeseumi	s639-1-1
	14	qBsmc14-2	3.99	10.32	0.74	s639-1-1~s1685-1	Yeseumi	s639-1-1
	14	qBsmc14-3	3.03	4.59	0.01	s277-2~s429-2	Yeseumi	s429-2
Secondary color of outer skin (△E)	6	qBssc6	3.49	3.79	0.01	s1680-1~s571-2	Yeseumi	s571-2
	13	qBssc13	3.22	5.95	0.54	s678-2~s42-4	Yeseumi	s42-4

^a^ Add.: Positive values for additive effect indicate that alleles from ‘Yeseumi’ are in the direction of increasing the traits.

^b^ R2: The proportion of evaluated phenotypic variation attributable to a particular QTL was estimated by the coefficient of determination.

^c^ Marker bordering the QTL: Markers within the significance threshold on each border of the QTL range.

^d^ Increasing effect: The increase allele is the source of the allele causing an increase in the measured trait.

## Supporting information

S1 File **Combined supporting information file–**Table A and Figure A-X.
(DOCX)
