# Supplementary material for: Construction of a genetic map using EST-SSR markers and QTL analysis of major agronomic characters in hexaploid sweet potato (Ipomoea batatas (L.) Lam)
Source: PLoS One. 2017 Oct 11;12(10):e0185073. doi: 10.1371/journal.pone.0185073 (PMC5636084; doi:10.1371/journal.pone.0185073)
Supplement: S1 File — Combined supporting information file–Table A and Figure A-X. (DOCX) [file pone.0185073.s001.docx]

**Table A. Characteristics of sweet potato**

| **Item** | **Character** | |
| --- | --- | --- |
| Above-ground part | Stem | length of internode, internode diameter, degree of anthocyanin expression in terminal bud, degree of anthocyanin expression in burl |
|  | Leaf | number of lobes per leaf, unlobed leaf shape, depth of leaf lobes |
| Underground part | Tuber | tuber form, length to width ratio, skin thickness, main color of outer skin, secondary color of outer skin, main color of flesh, secondary color of flesh, depth of tuber bud |


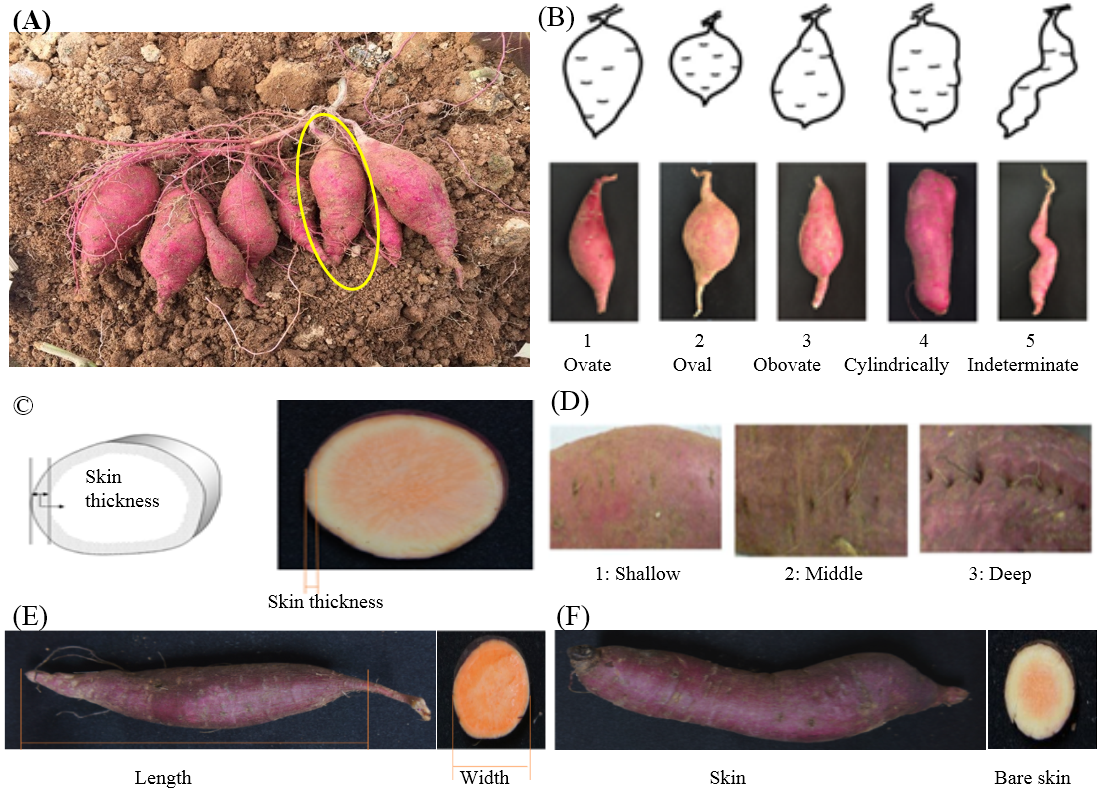


**Figure A.** Underground part. **(**A): **y**ellow color circle was selected and examined, (B): root form, (C): skin thickness (mm), (D): depth of root buds, (E): ration of length to with at root, (F): measurement of color at skin and bare skin.


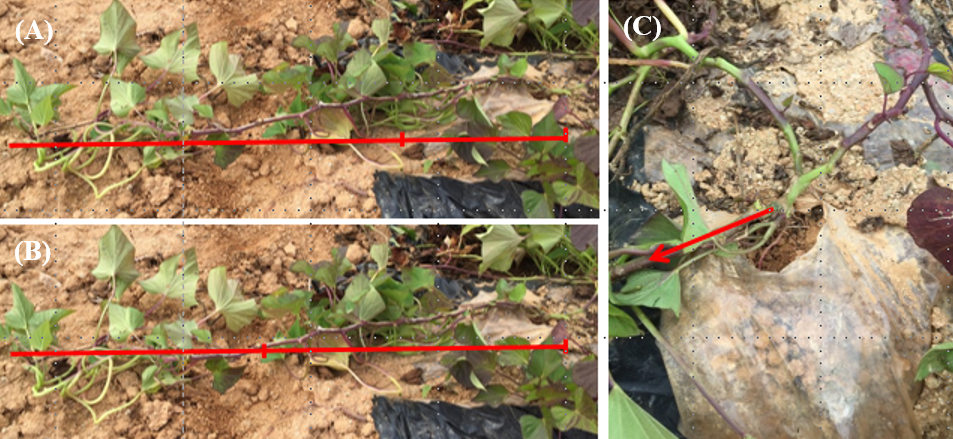


**Figure B.** Internode length. (A): the red line shows the one-third point, (B): the red line shows the midpoint, (C): the red arrow indicates the main stem.


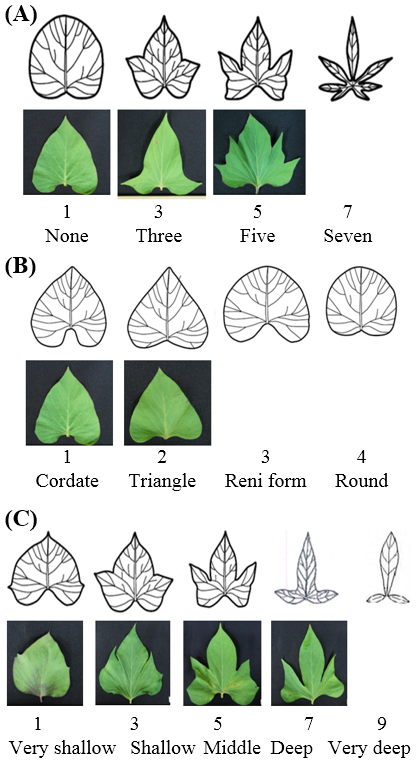


**Figure C.** Leaf characteristics. (A): number of leaf lobes, (B): unlobed leaf shape, (C): leaf lobe depth.


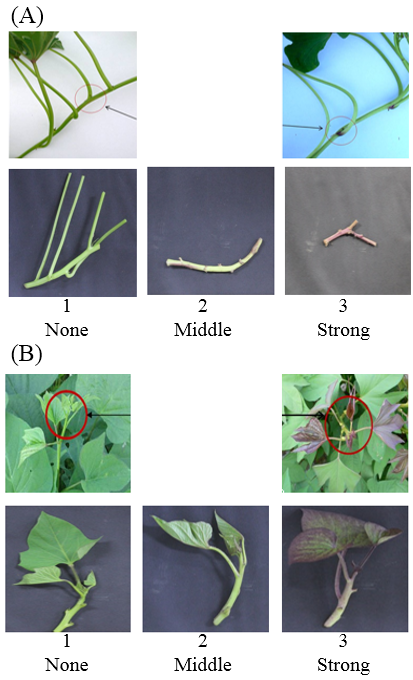


**Figure D.** Stem characteristics. (A): degree of anthocyanin biosynthesis in burl, (B): degree of anthocyanin biosynthesis in terminal bud.


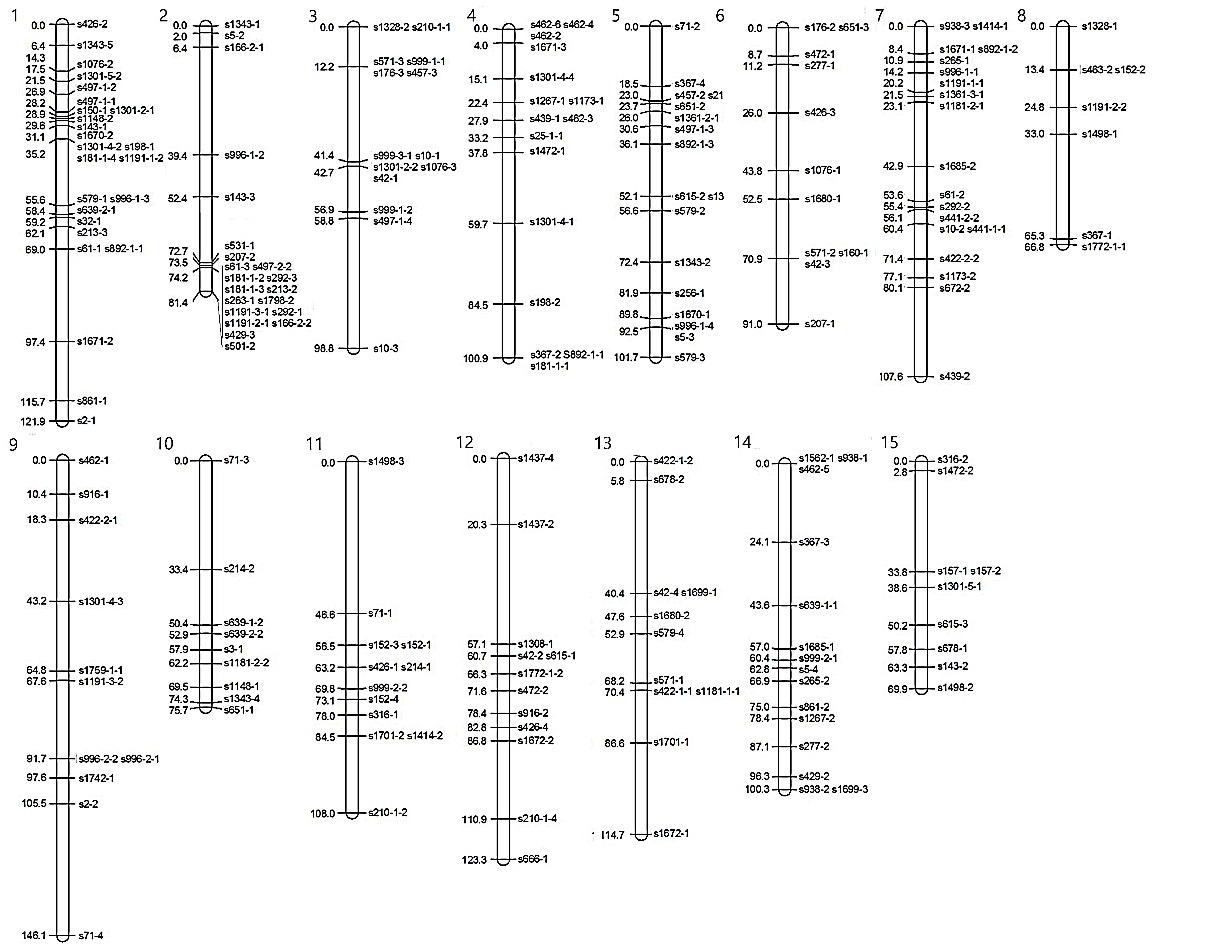


**Figure E.** Genetic-linkage grouping of 137 lines derived from a cross between Yeseumi and Annobeny.


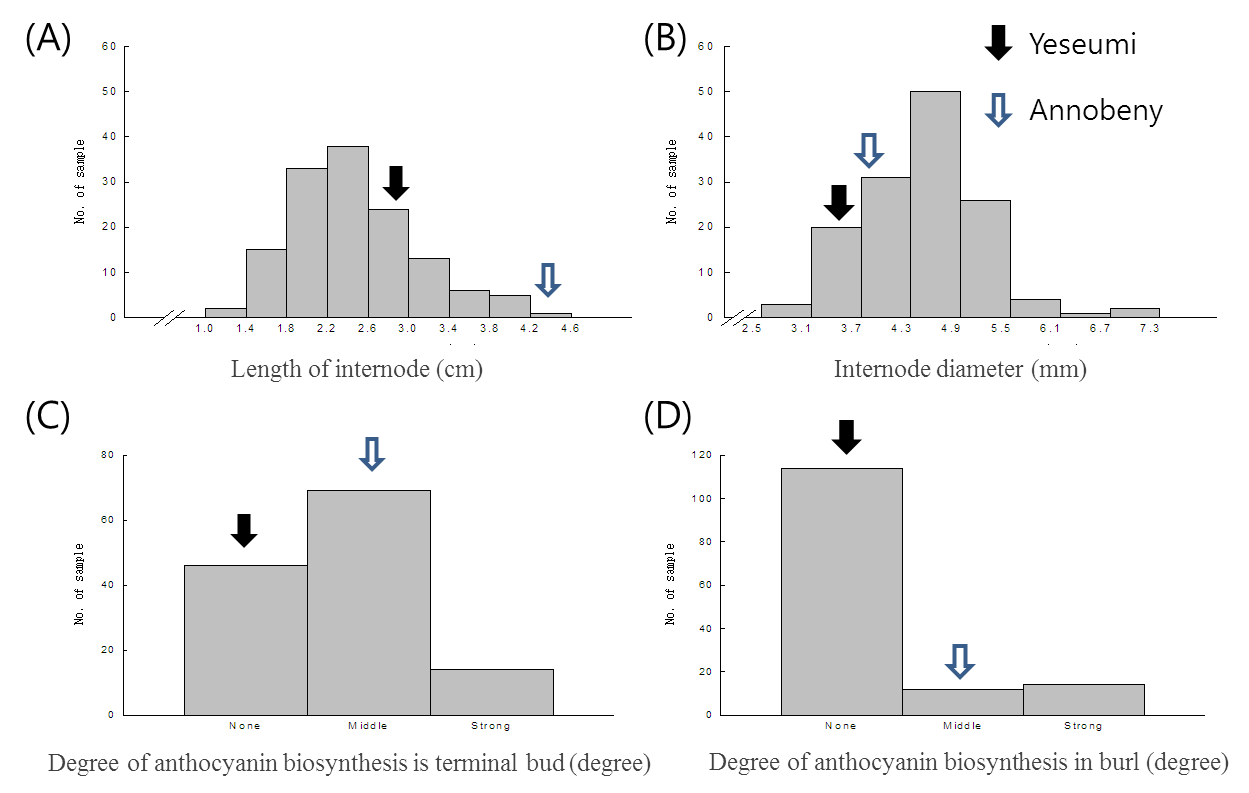


**Figure F.** Distribution of in the population of sweetpotato from a cross of Yeseumi/Annobeny. The black arrow represents Yeseumi while the white arrow represents Annobeny. (A): length of internode (cm), (B): internode diameter (mm), (C): degree of anthocyanin biosynthesis is terminal bud, (D): degree of anthocyanin biosynthesis in burl.


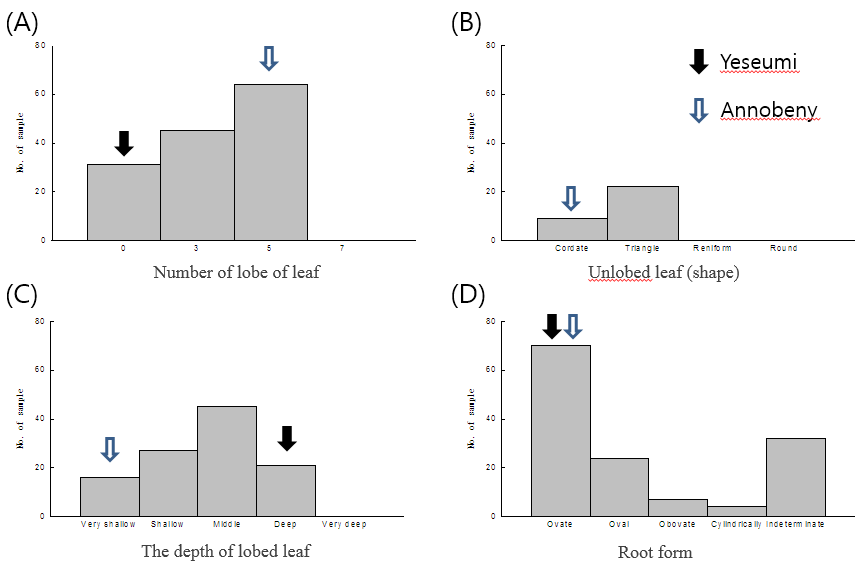


**Figure G.** Distribution of in the population of sweetpotato from a cross of Yeseumi/Annobeny. The black arrow represents Yeseumi while the white arrow represents Annobeny. (A): number of lobe of leaf, (B): unlobed leaf (shape), (C): the depth of lobed leaf, (D): root form.


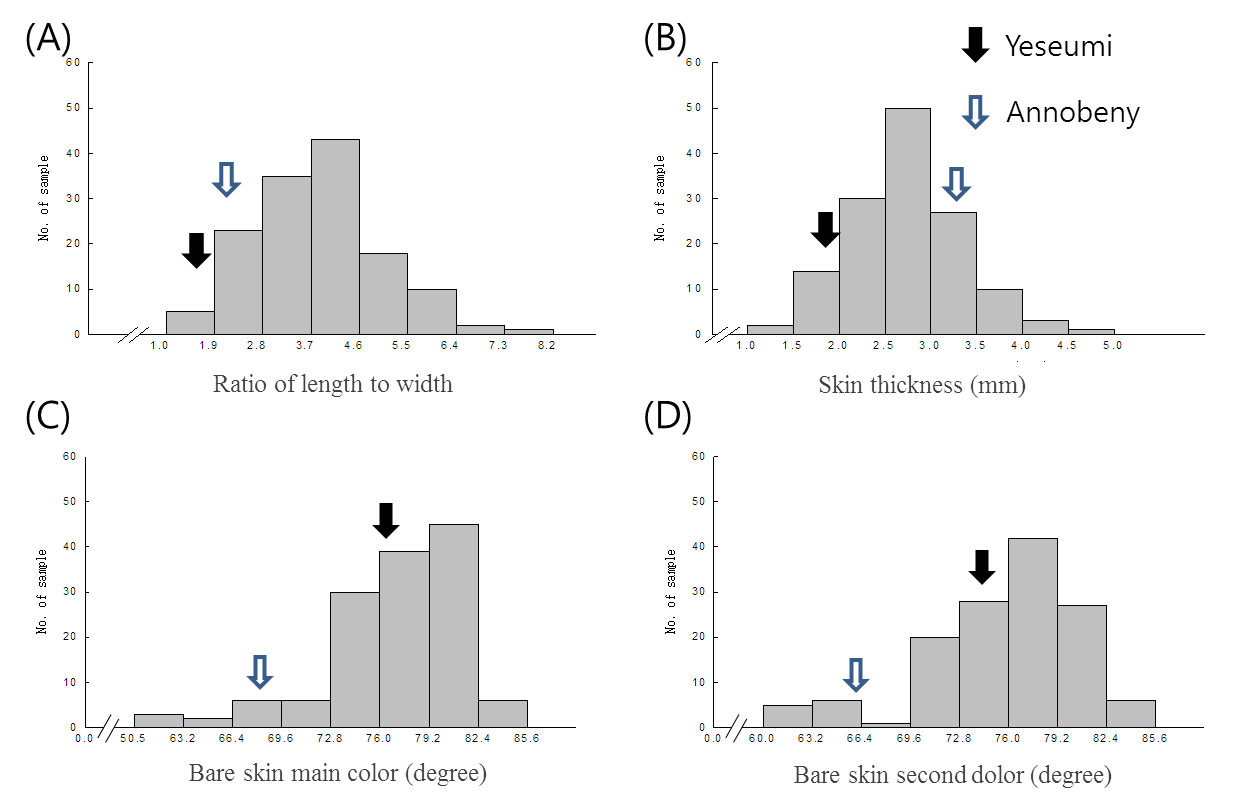


**Figure H.** Distribution of in the population of sweetpotato from a cross of Yeseumi/Annobeny. The black arrow represents Yeseumi while the white arrow represents Annobeny. (A): ratio of length to width, (B): skin thickness (mm), (C): bare skin main color (△E), (D): bare skin second color (△E).


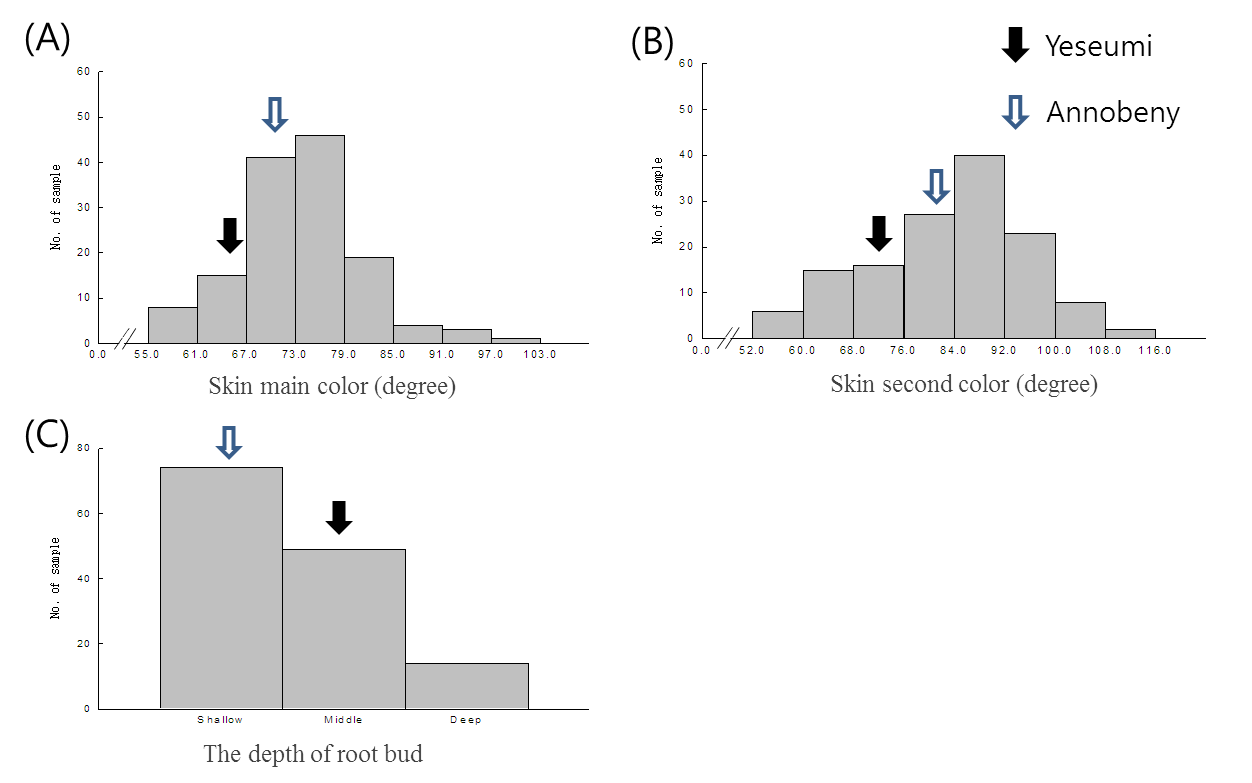


**Figure I.** Distribution of in the population of sweetpotato from a cross of Yeseumi/Annobeny. The black arrow represents Yeseumi while the white arrow represents Annobeny. (A): skin main color (△E), (B): skin second color (△E), (C): the depth of tuber bud.


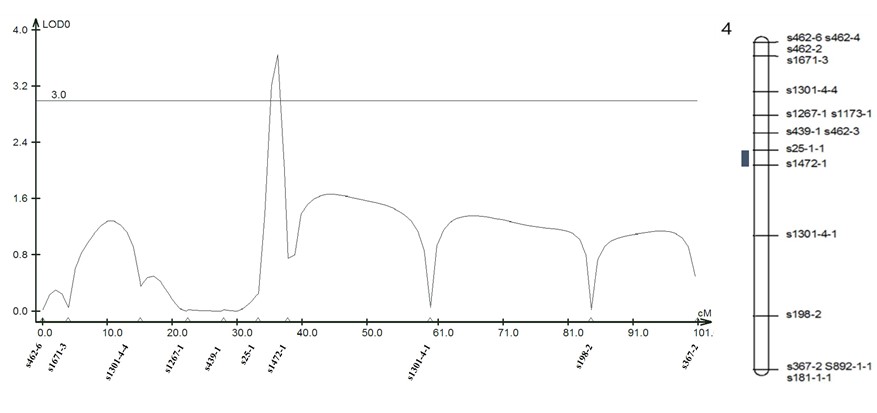


**Figure J.** The chromosomal location of QTL associated with length of internode in Yesemi and Annobeny population on group 4.


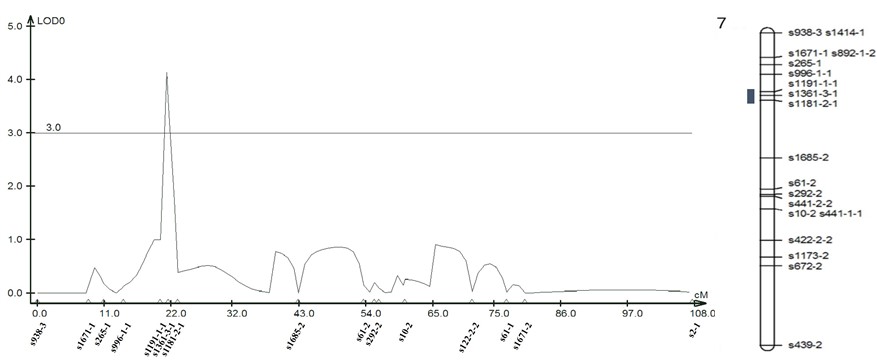


**Figure K.** The chromosomal location of QTL associated with length of internode in Yesemi and Annobeny population on group 7.


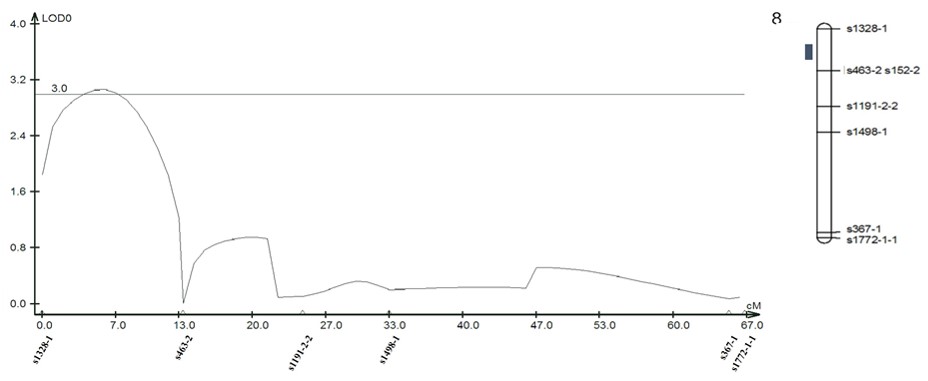


**Figure L.** The chromosomal location of QTL associated with length of internode in Yesemi and Annobeny population on group 8.


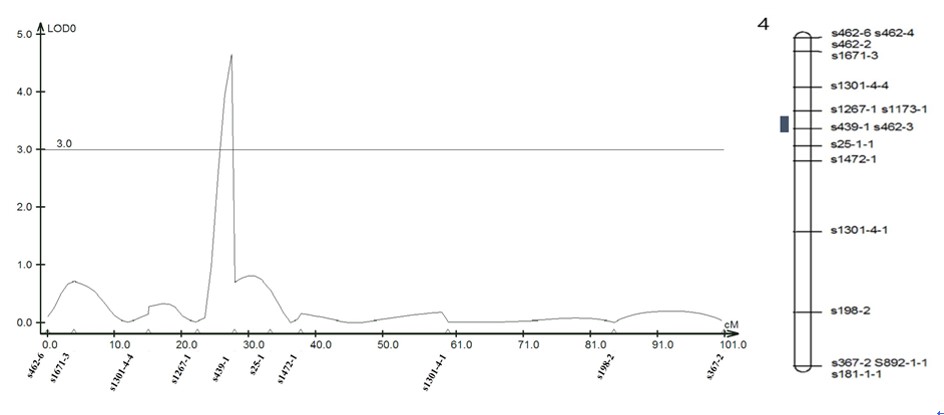


**Figure M.** The chromosomal location of QTL associated with skin thickness in Yeseumi and Annobeny population on group 4.


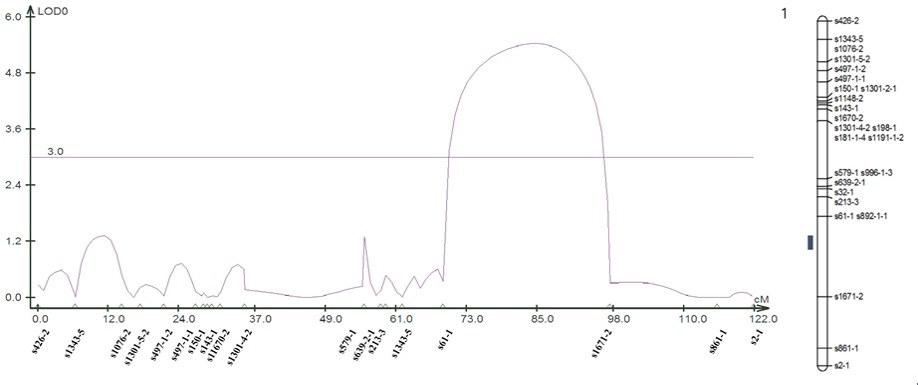


**Figure N.** The chromosomal location of QTL associated with bare skin main color in Yeseumi and Annobeny population on group 1.


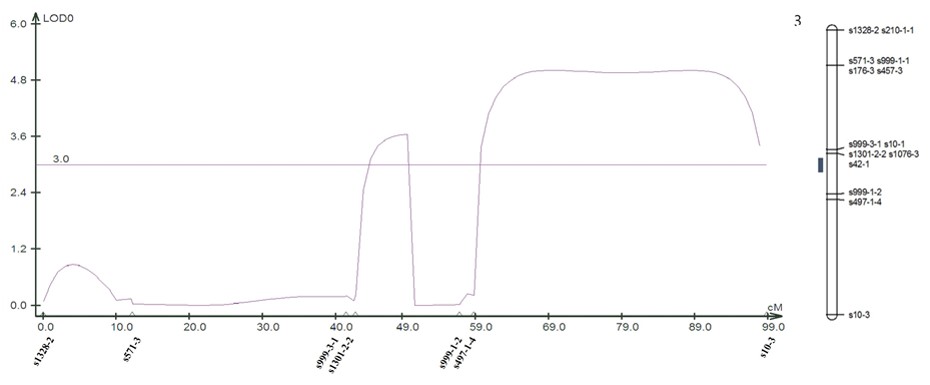


**Figure O.** The chromosomal location of QTL associated with bare skin main color in Yeseumi and Annobeny population on group 3.


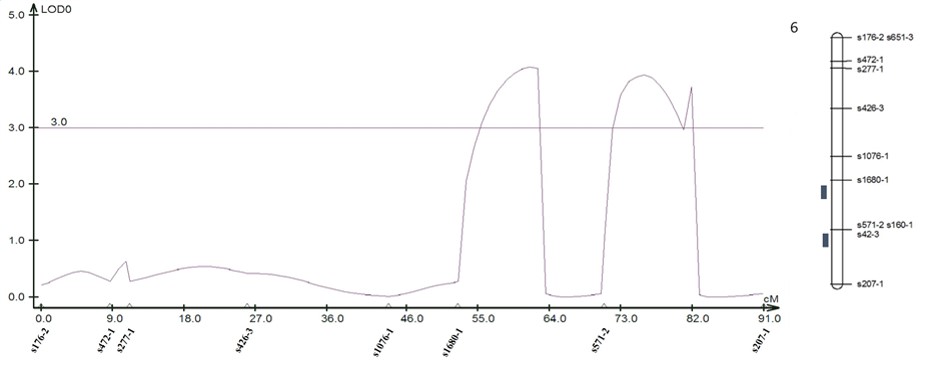


**Figure P.** The chromosomal location of QTL associated with bare skin main color in Yeseumi and Annobeny population on group 6.


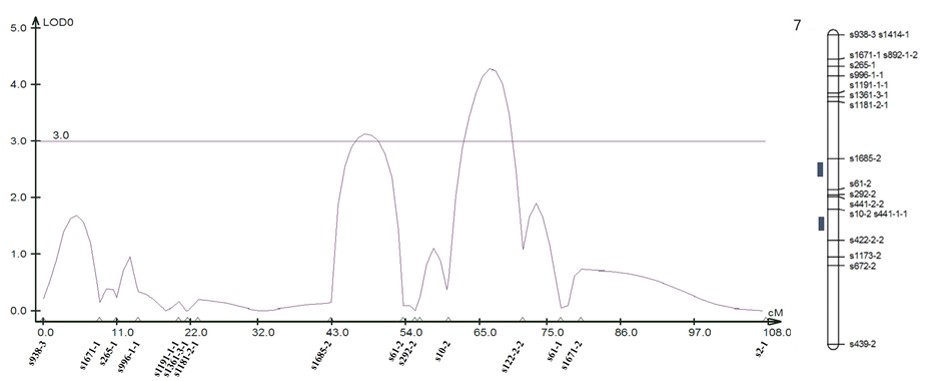


**Figure Q.** The chromosomal location of QTL associated with bare skin main color in Yeseumi and Annobeny population on group 7.


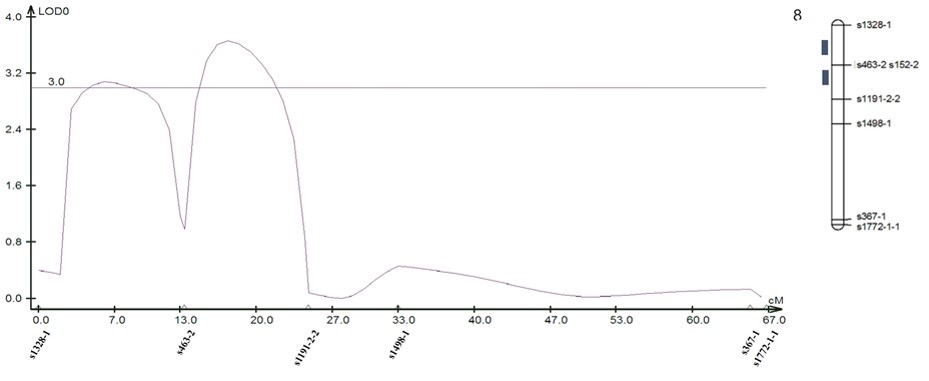


**Figure R.** The chromosomal location of QTL associated with bare skin main color in Yeseumi and Annobeny population on group 8.


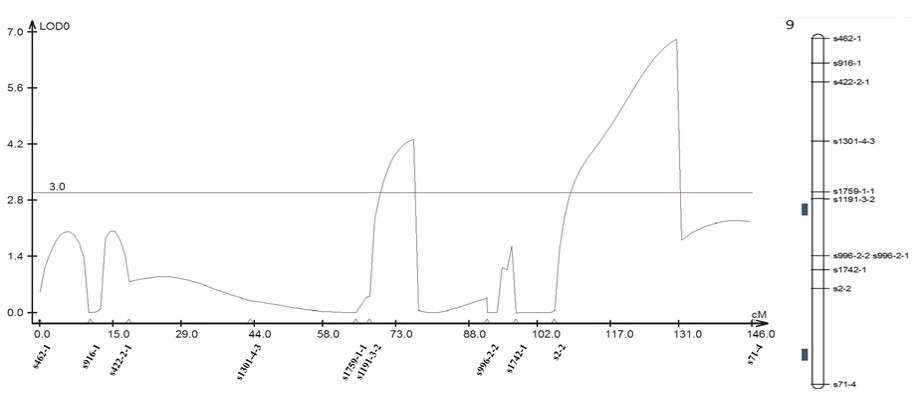


**Figure S.** The chromosomal location of QTL associated with bare skin main color in Yeseumi and Annobeny population on group 9.


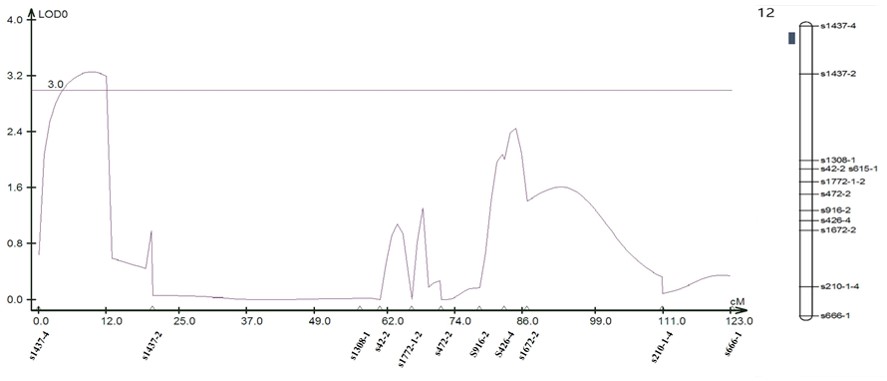


**Figure T.** The chromosomal location of QTL associated with bare skin main color in Yeseumi and Annobeny population on group 12.


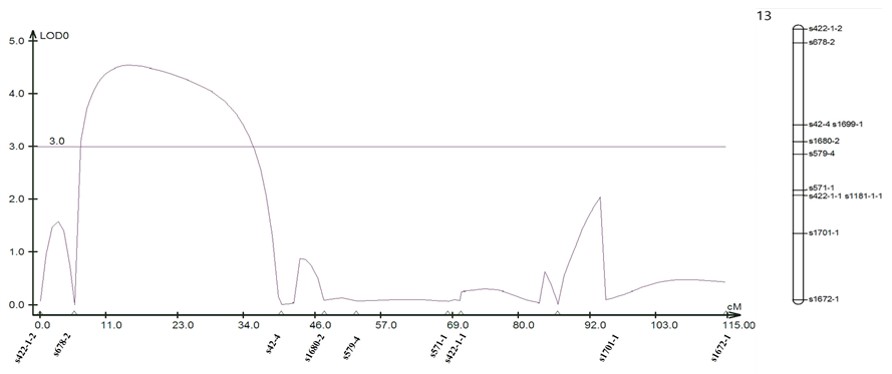


**Figure U.** The chromosomal location of QTL associated with bare skin main color in Yeseumi and Annobeny population on group 13.


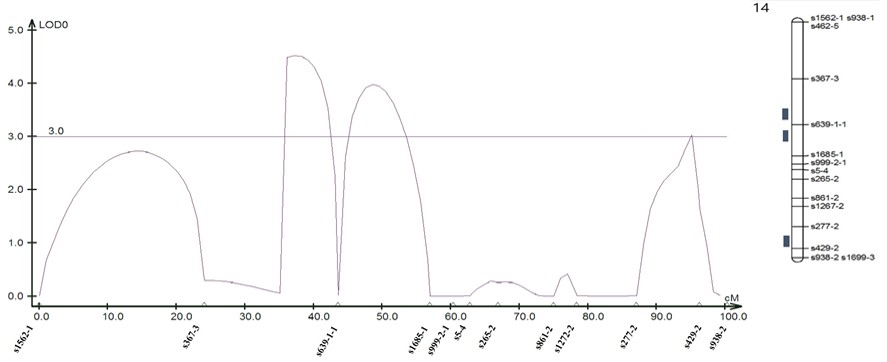


**Figure V.** The chromosomal location of QTL associated with bare skin main color in Yeseumi and Annobeny population on group 14.


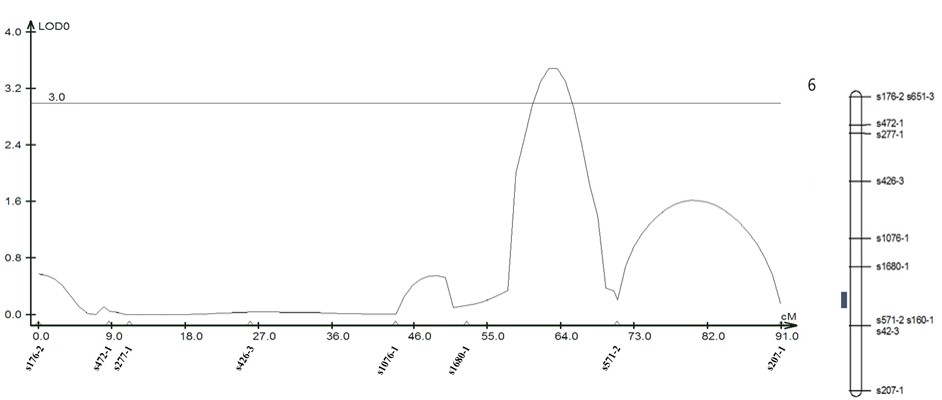


**Figure W.** The chromosomal location of QTL associated with bare skin second color in Yeseumi and Annobeny population on group 6.


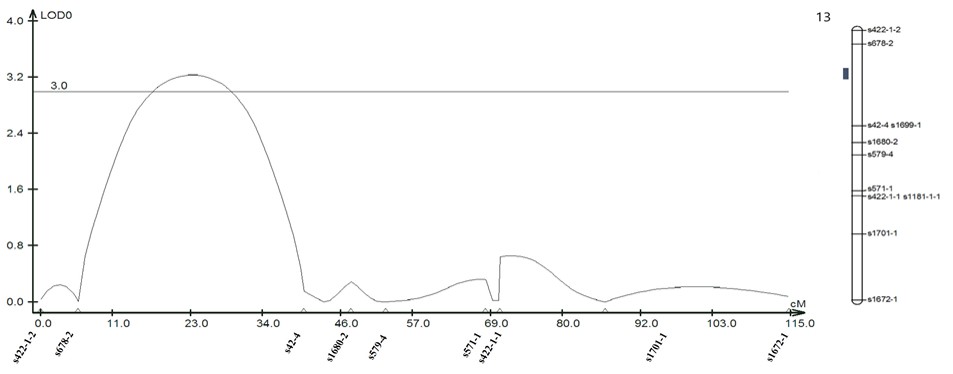


**Figure X.** The chromosomal location of QTL associated with bare skin second color in Yeseumi and Annobeny population on group 13.
